# Curcumin induces apoptosis-independent death in oesophageal cancer cells

**DOI:** 10.1038/sj.bjc.6605308

**Published:** 2009-10-06

**Authors:** G O'Sullivan-Coyne, G C O'Sullivan, T R O'Donovan, K Piwocka, S L McKenna

**Affiliations:** 1 Leslie C. Quick Laboratory, Cork Cancer Research Centre, BioSciences Institute, University College Cork and Mercy University Hospital, Cork, Ireland; 2 Nencki Institute of Experimental Biology, Warsaw, Poland

**Keywords:** curcumin, apoptosis, mitotic catastrophe, autophagy, oesophageal cancer

## Abstract

**Background:**

Oesophageal cancer incidence is increasing and survival rates remain extremely poor. Natural agents with potential for chemoprevention include the phytochemical curcumin (diferuloylmethane). We have examined the effects of curcumin on a panel of oesophageal cancer cell lines.

**Methods:**

MTT (3-(4,5-dimethyldiazol-2-yl)-2,5 diphenyl tetrazolium bromide) assays and propidium iodide staining were used to assess viability and DNA content, respectively. Mitotic catastrophe (MC), apoptosis and autophagy were defined by both morphological criteria and markers such as MPM-2, caspase 3 cleavage and monodansylcadaverine (MDC) staining. Cyclin B and poly-ubiquitinated proteins were assessed by western blotting.

**Results:**

Curcumin treatment reduces viability of all cell lines within 24 h of treatment in a 5–50 *μ*M range. Cytotoxicity is associated with accumulation in G2/M cell-cycle phases and distinct chromatin morphology, consistent with MC. Caspase-3 activation was detected in two out of four cell lines, but was a minor event. The addition of a caspase inhibitor zVAD had a marginal or no effect on cell viability, indicating predominance of a non-apoptotic form of cell death. In two cell lines, features of both MC and autophagy were apparent. Curcumin-responsive cells were found to accumulate poly-ubiquitinated proteins and cyclin B, consistent with a disturbance of the ubiquitin–proteasome system. This effect on a key cell-cycle checkpoint regulator may be responsible for the mitotic disturbances and consequent cytotoxicity of this drug.

**Conclusion:**

Curcumin can induce cell death by a mechanism that is not reliant on apoptosis induction, and thus represents a promising anticancer agent for prevention and treatment of oesophageal cancer.

Oesophageal cancer is the eighth most common cancer and the sixth most common cause of death from cancer (Parkin *et al*, 2005). Despite advances in surgery and neoadjuvant therapy, 5-year survival rates for oesophageal cancer are between 10 and 15% for the United States and 10% for Europe (Sant *et al*, 2003). The pathogenesis of this cancer still remains unclear, making the prediction of sensitivity to current chemotherapeutic regimes very difficult. The development of new agents for treatment of this cancer is imperative, requiring a more novel and integrated approach to prevention, diagnosis and treatment.

Tumour cell resistance to apoptosis is an inherent part of the carcinogenic process and is also implicated in resistance to chemotherapeutic drugs (Johnstone *et al*, 2002). Therefore, new approaches are required to target resistant cells and improve efficacy without toxicity. Recent attention has focused on phytochemicals as anticancer agents. Curcumin is a yellow pigment found in the rhizome of turmeric (*Curcuma longa*), and has been attributed to various properties including, antioxidant, anti-inflammatory, anti-angiogenic, anti-proliferative and wound healing (Sharma *et al*, 2005), without cytotoxic effects on healthy cells (Syng-Ai *et al*, 2004). It has also shown significant chemopreventive efficacy against various malignancies (Surh, 2003). Several studies have suggested that curcumin induces apoptotic cell death in malignant cells (Karunagaran *et al*, 2005) and to be both p53-dependent and p53-independent (Salvioli *et al*, 2007). The exact mechanisms of curcumin-induced cell death still remain ill-defined, with controversial roles for the Bcl-2 family and reactive oxygen species (Salvioli *et al*, 2007).

There is also growing evidence that curcumin can induce death in cells that are resistant to apoptosis (Holy, 2002; Wolanin *et al*, 2006). Several studies have found that curcumin induces arrest within the G2/M phases of the cell cycle, together with mitotic spindle disruption, defects in cytokinesis and micronucleation, consistent with a type of cell death referred to as mitotic catastrophe (MC) (Magalska *et al*, 2006; Wolanin *et al*, 2006). More recently, treatment with curcumin has been reported to induce autophagic cell death in malignant cells (Aoki *et al*, 2007). This type of cell death occurs without chromatin fragmentation, and is characterised by the formation of autophagic vacuoles (Degterev and Yuan, 2008).

In accordance with the multiple properties and clinical effects ascribed to curcumin, many cellular targets have also been proposed. Curcumin has been reported to inhibit diverse targets such as NF-κB, COX-2 and kinases associated with survival signalling (IKK, NIK and AKT), cell proliferation (ERK) and cell-cycle regulation (Aggarwal *et al*, 2003; Castedo *et al*, 2004; Duvoix *et al*, 2005). More recently, downregulation of the survivin protein has been implicated in curcumin-induced MC (Magalska *et al*, 2006; Wolanin *et al*, 2006).

The ability of curcumin to show a broad range of activity has been attributed to its ability to affect multiple targets. However, how curcumin actually achieves this broad-spectrum activity is unknown. In this study, we have investigated whether curcumin could have cytotoxic activity against oesophageal cancer cells. It is likely that this is one of the most suitable cancers for oral administration of curcumin, because of low bioavailability for other non-gastrointestinal cancers, and the increasing diagnosis worldwide of pre-malignant conditions such as Barrett's oesophagus.

As oesophageal cancers are often resistant to cell death induction with chemotherapeutic drugs, we have specifically examined how curcumin induces cell death, and looked for molecular features that are associated with susceptibility to this agent. We have shown that curcumin primarily induces MC in susceptible cells. In addition, apoptosis or autophagy can accompany MC – and this is probably dependent on the active death signalling pathway in a given cell line. We have also examined additional molecular effects of curcumin that could potentially account for both its pleiotropic actions and the mitotic death mechanism. Cytotoxicity correlated with the ability of curcumin to elevate the levels of poly-ubiquitinated proteins and to accumulate the mitotic regulator cyclin B1. This is consistent with evidence that curcumin can influence multiple targets because of its inhibitory effects on the ubiquitin–proteasome system (UPS) (Jana *et al*, 2004; Si *et al*, 2007).

## Materials and methods

### Cell lines and culture conditions

Human oesophageal squamous and adenocarcinoma cell lines OE19, OE21 and OE33 (Rockett *et al*, 1997) were purchased from the European Collection of Cell Cultures (ECACC). KYSE450 were purchased from Die Deutsche Managementsystem Zertifizierungsgesellschaft mbH (DSMZ, Braunschweig, Germany). The cells were maintained in RPMI 1640, 1% penicillin/streptomycin, 10% (v/v) fetal calf serum (FCS, all from Gibco, Paisley, UK) at 37°C in 95% air and 5% CO_2_.

### Cell treatment

Curcumin (Cayman Chemical Co., Ann Arbor, MI, USA) was dissolved in dimethyl sulphoxide (DMSO) (Sigma, Dublin, Ireland). Final curcumin concentrations of 5–50 *μ*M were obtained by dilution in culture media such that the final concentration of DMSO was not >0.07%. Controls containing 0.07% DMSO were included in all experiments. For inhibition of caspase activity, cells were treated with Z-Val-DL-Asp-fluoromethylketone (zVAD-fmk) (Bachem AG, Bubendorf, Switzerland) at a concentration of 25 or 50 *μ*M for 1 h before curcumin treatment. Cells were treated with 200 nM of nocodazole for 18 h before MPM-2 expression assay.

### Proliferation and viability assays

Cell viability was measured by MTT ((3-(4,5-dimethyldiazol-2-yl)-2,5 diphenyl tetrazolium bromide); Sigma). After 24 h treatment, MTT (5 mg ml^−1^ in phosphate buffered saline (PBS)) was added to each well and incubated for 1 h at 37°C in 95% air and 5% CO_2_. Reduced MTT was dissolved in DMSO and measured spectrophotometrically in a dual-beam microtiter plate reader (SpectraMax, Molecular Devices, Sunnyvale, CA, USA) at 562 with a 620-nm reference using SoftMax Pro 4.7.1 software (Molecular Devices). Experiments were carried out in quadruplicate wells, repeated at least three times. Values are presented as the mean absorbance±s.e.m.

### Cell cycle analysis

After 24 h of treatment with curcumin (5–50 *μ*M), cells were harvested and fixed in 70% ethanol overnight at 4°C. Cells were washed twice in PBS, and resuspended in 500 *μ*l of PBS with 20 *μ*g ml^−1^ propidium iodide (PI) (Sigma, Dublin, Ireland) for 30 min in the dark. Cell cycle was analysed by flow cytometry using CellQuest software Becton Dickinson, Oxford, UK on a FACScan Flow Cytometer (Becton Dickinson, Oxford, UK).

### Cell morphology

Cell suspensions (100 *μ*l) were cytospun in a Shandon Cytospin 4 Cytocentrifuge (Thermo Scientific, Cheshire, UK) at 400 r.p.m. for 2 min, air-dried and stained using RapiDiff (Bios Europe, Lancashire, UK). Slides were mounted with DPX (BDH Laboratories, Poole, UK) and visualised using Olympus BX51 system DP70 camera with Olympus DP3.2 software (Essex, UK).

### Mitotic index analysis

The mitotic index was assessed by MPM-2 (anti-phospho-Ser/Thr-Pro) expression. After 24 h of treatment (15–25 *μ*M curcumin), cells were harvested and fixed in 70% ethanol overnight. Cells were then washed and suspended in 100 *μ*l of IFA-Tx buffer (4% FCS, 150 nM NaCl, 10 nM HEPES, 0.1% sodium azide, 0.1% Triton X-100) with a primary MPM-2 antibody (1 *μ*g ml^−1^; Upstate Cell Signaling Solutions, Millipore, Watford, UK) at room temperature for 1 h. Cells were washed and resuspended in IFA-Tx buffer with a rabbit anti-mouse FITC-conjugated secondary antibody (1 : 50; Serotec, Oxford, UK) for 1 h at room temperature in darkness. Finally, cells were washed and resuspended in 500 *μ*l of PBS with 20 *μ*g ml^−1^ of PI (Sigma) for 30 min in the dark. MPM-2 expression was analysed using CellQuest software on a FACScan flow cytometer (Becton Dickinson).

### Western blot analysis

Cells were washed in ice-cold PBS (Gibco) and lysed in ice-cold RIPA buffer (50 mM Tris-HCl (pH 7.4), 1% Igepal, 0.25% sodium deoxycholate, 150 mM NaCl, 1 mM EGTA, 1 mM Na_3_VO_4_, 1 mM NaF) with the addition of 1 × protease inhibitor cocktail (Sigma) and 1 × Pefabloc SC (Fluka, Buchs SG, Switzerland) before use. Protein estimation of supernatants was carried out using the Bradford assay. Equal concentrations (50 *μ*g) of protein were separated by SDS–PAGE, transferred to Protran nitrocellulose membrane (Perkin-Elmer, Boston, MA, USA) and blocked in 5% non-fat milk in PBS. Membranes were probed with polyclonal anti-caspase3 antibody at 1 : 1000 (Cell Signaling Technology Inc., Danvers, MA, USA). For the detection of phospho-p44/42 MAP kinase, membranes were probed with ERK and P-ERK polyclonal rabbit antibodies (1 : 1000; Cell Signaling Technology Inc.). Other antibodies were mouse monoclonal anti-survivin antibody (dilution at 1 : 100; Santa Cruz Biotechnology Inc., Santa Cruz, CA, USA), rabbit polyclonal anti-ubiquitin antibody (1 : 1000; Cell Signaling Technology Inc.) and rabbit polyclonal anti-cyclin B1 (1 : 500; Santa Cruz Biotechnology Inc). All secondary antibodies were horseradish peroxidase-conjugated and used at 1 : 1000 (DakoCytomation, Cambridgeshire, UK). Peroxidase activity was detected using an enhanced chemiluminescence detection kit (Amersham Biosciences, Buckinghamshire, UK) and developed on Kodak film (Sigma). Membranes were re-probed with anti-*β*-actin antibody 1 : 5000 (Sigma).

### Detection of acidic autophagic vacuoles

Cells were seeded on sterile coverslips in tissue culture plates (Cover Glasses, Menzel-Glaser, Germany). After curcumin treatment, cells were incubated with 0.1 mM monodansylcadaverine (MDC) in PBS at 37°C for 10 min, washed thrice with PBS and immediately visualised by fluorescent microscopy using an inverted microscope and an Olympus BX51 system DP70 camera. Excitation wavelengths were 360–380 nm. For analysis and photography, Olympus DP version 3.2 software were used.

### Statistical analysis

Comparison between groups was carried out with paired Student's *t*-test. Values of *P*<0.05 were considered statistically significant. Asterisks indicate the level of significance.

## Results

### Effects of curcumin on cell viability and clonogenicity in oesophageal cancer cells

**Figure 1:**
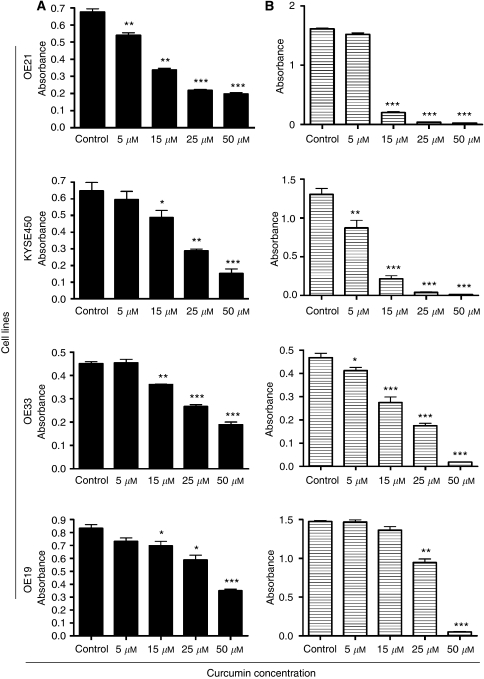
Assessment of sensitivity and clonogenicity of squamous cell carcinoma and adenocarcinoma oesophageal cell lines to curcumin. (**A**) Effects of curcumin on cell viability. After treatment with 5, 15, 25 and 50 *μ*M of curcumin for 24 h, cell viability was determined by MTT assay, with values presented as mean absorbance. ^*^P<0.05, ^**^P<0.005 and ^***^P<0.0005 compared with untreated cells (paired Student's *t*-test). Bars=s.e.m. (**B**) To determine whether or not the cells could overcome the effects of the drug and recover, the curcumin was removed after 24 h of treatment. Cells were allowed to recover for a further 48 h and the MTT assay was repeated. Cell clonogenicity was calculated as the percentage of the control. ^*^P<0.05, ^**^P<0.005 and ^***^P<0.0005 compared with untreated cells (paired Student's *t*-test). Bars=s.e.m.

We assessed the sensitivity of a panel of oesophageal cancer cell lines to curcumin. This includes two squamous cell carcinoma (SCC) cell lines (OE21 and KYSE450) and two adenocarcinoma cell lines (AC) (OE33 and OE19). All cell lines were treated with 5, 15, 25 and 50 *μ*M of curcumin for 24 h. Curcumin induced a dose-dependent reduction in viability in all cell lines, as measured by the MTT assay (Figure 1A). However, the OE19 cell line was less affected at lower concentrations.

As the MTT assay evaluates a metabolic activity, we further assessed whether viability was indeed lost by assessing the capacity of the cultures to recover from treatment after removal of curcumin. Cells were incubated with curcumin for 24 h and then allowed to recover without drug for a further 48 h. Concentrations in excess of 5 *μ*M markedly reduced the recovery of all cell lines, indicating substantial loss of viability. The OE19 cells showed greater resistance and were better able to recover at 15 and 25 *μ*M concentrations (Figure 1B). According to the MTT assay, the cell lines can be ranked in the following order regarding their ability to recover after curcumin treatment: KYSE450>OE21>OE33>OE19. The KYSE450 and OE21 cell lines cannot recover from curcumin treatment at concentrations higher than 15 *μ*M.

### Effect of curcumin on the cell cycle and mitotic index

**Figure 2:**
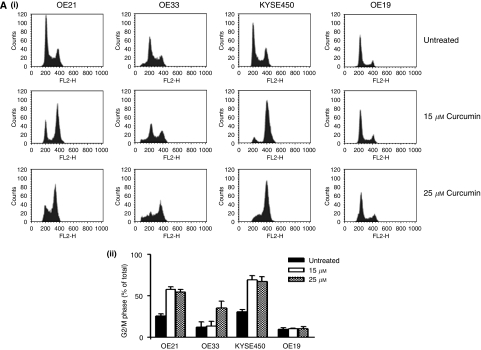

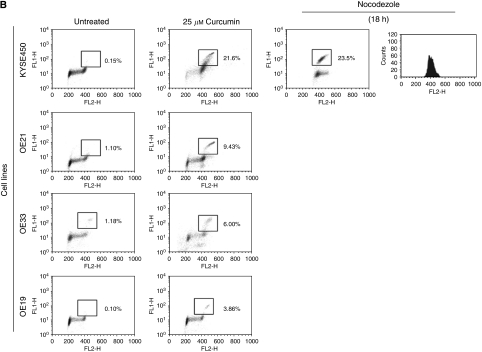
(**A**) (i) Assessment of DNA content in oesophageal cancer cell lines after 24 h of treatment with curcumin. DNA content analysis was carried out by propidium iodide staining and flow cytometry after 24 h of treatment with increasing concentrations of curcumin. A total of 10 000 cells were counted for each cell line and concentration. Images correspond to typical histogram distributions for each cell line at 0, 15, 25 *μ*M of curcumin. The percentage of cells in the G2/M phase of the cell cycle was estimated using CellQuest software. (ii) The percentage of cells arrested in G2/M phase of the cell cycle. Columns are means of three experiments. Bars=s.d. (**B**) MPM-2 (anti-phospho-Ser/Thr-Pro) expression in untreated and treated oesophageal cancer cells. MPM-2 is an antibody that recognises a group of proteins that are phosphorylated only in mitosis. Cells were dually stained with propidium iodide to analyse DNA content, and expression was quantified by flow cytometry. As a positive control, KYSE450 cells were treated for 18 h with nocodazole, an anti-fungal agent known to induce metaphase arrest. Cell-cycle analysis and quantification of MPM-2 expression (gated cells) were carried out by flow cytometry after treatment with 25 *μ*M of curcumin for 24 h.

We then examined cell-cycle distribution for each of the cell lines after treatment with a range of curcumin concentrations (Figure 2Ai). These data show G2/M accumulation with increasing concentrations of curcumin in three out of four cell lines. The more resistant OE19 cell line failed to show G2/M accumulation at the concentrations shown. Interestingly, the most sensitive cell lines (KYSE450 and OE21), which have undergone significant cell death in the 15–50 *μ*M range, do not show significant sub-G1 DNA content, which would be typical if apoptosis was induced. The percentage of cells in G2/M phases of the cell cycle increased with higher concentrations of curcumin (Figure 2Aii) in all three sensitive cell lines.

In order to distinguish G2 arrest from mitotic arrest, we also examined an additional marker. MPM-2 is an antibody that recognises a group of proteins whose epitopes are exclusively phosphorylated during mitosis – particularly early prophase to metaphase (Davis *et al*, 1983) and it is commonly used as an indicator of mitotic disturbance. As a positive control, cells were treated with nocodazole, a known inducer of metaphase arrest (Vassilev *et al*, 2006). Treatment of KYSE450 cells with nocodazole for 18 h resulted in a synchronisation of the entire cell population in the G2/M phase and an increase in MPM-2 labelling from 0.15 to 23.5% (Figure 2B). In all cells treated with 25 *μ*M of curcumin, MPM-2 staining was elevated above control levels (21.61, 9.43, 6 and 3.86% for KYSE450, OE21, OE33 and OE19, respectively) (Figure 2B). Staining was not as strong as that achieved with nocodazole, possibly because the G2M accumulation was not as marked (except in KYSE450 cells). It is also likely that the cells are in various stages of mitosis, which are not all identified with this early prophase marker. Therefore, the elevated staining of MPM-2 suggests mitotic disturbance – but may also underestimate it.

### Morphological analysis of oesophageal cancer cells after 24 h of curcumin treatment

**Figure 3:**
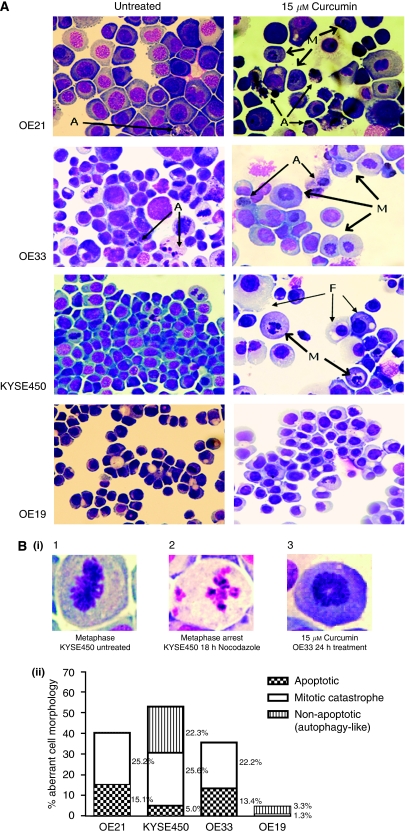
Morphological features of curcumin-induced cell death in oesophageal cancer cell lines. Cell morphology was visualised using RapiDiff staining by light microscopy after treatment with 15 *μ*M of curcumin for 24 h. (**A**) Typical cytospin images for untreated and curcumin-treated oesophageal cancer cell lines. Curcumin treatment (15 *μ*M) produced a distinct morphology suggestive of a monopolar spindle and duplicated but unseparated chromosomes centrally located in the cell (M); features are consistent with chromatin images described for mitotic catastrophe (MC). In addition to MC, OE21 and OE33 cell lines show a minor population of cells with clear apoptotic morphology (A). Both of these cell lines also have a background of apoptotic cells visible in their untreated cytospins. By contrast, KYSE450 and OE19 cell lines rarely show apoptotic cells. KYSE450-treated cells predominantly show the typical nuclear morphological features of MC after curcumin treatment. Other features coexist at a background level, including nuclear pyknosis, vacuolisation of the cytoplasm or complete loss of the cytoplasmic membrane while the nuclear membrane remains intact; elements consistent morphologically with autophagic cell death (F). OE19-treated cells preserve their overall structural features at this concentration. (**B**) (i) Typical chromatin organisations in untreated and treated cells. (1) Normal mitosis metaphase image with chromosomes along the equatorial plane of the cell. (2) Nuclear features of cells treated with 18 h of nocodazole, with typical micronucleation. (3) Chromatin alignment in cells treated with curcumin for 24 h. (ii) Distribution of abnormal morphological features in RapiDiff-stained slides in all four oesophageal cancer cell lines after treatment with 15 *μ*M of curcumin over 24 h. Cells were counted on the basis of their morphology (apoptotic, non-apoptotic/MC and autophagy-like) and expressed as the percentage of the total cell population.

Curcumin susceptibility in these oesophageal cells is associated with induction of a cell-cycle arrest within the G2/M stages and mitotic disturbance. Other studies have reported that curcumin can induce MC, which is currently defined primarily by morphology (Kroemer *et al*, 2009). We therefore examined the morphology of curcumin-treated cells. Figure 3A shows representative examples of both treated and untreated cell lines. After 24 h treatment with concentrations as low as 15 *μ*M, a marked elevation in the number of cells with visibly distinct chromosomes was observed. Many showed a distinct chromatin image suggestive of a monopolar spindle and duplicated but unseparated chromosomes, which are centrally located in the cells (Figure 3A–M). In most cells, the nuclear membrane is absent and cells appear unable to progress beyond metaphase. In addition, some large cells with an extra nuclei were observed. These distinct morphologies were present and predominant in the three sensitive cell lines.

In addition to features of MC, the curcumin-treated OE21 and OE33 cell lines also show a subpopulation of cells with clear classical apoptosis morphology: chromatin condensation, cytoplasm shrinkage, and the formation of apoptotic bodies (Figure 3A (A)). Untreated control OE21 and OE33 cell lines also have a minor background of apoptotic cells. Apoptotic cells were very rarely found in the KYSE450 or OE19 population. After treatment with 15 *μ*M of curcumin, KYSE450 cells predominantly show MC, but other morphological features also coexist in the cell population. These include nuclear pyknosis and vacuolisation of the cytoplasm. OE19 cells treated with the same concentration of curcumin preserve their overall structural features similar to untreated cells. Much higher concentrations (⩾50 *μ*M) show similar morphology to KYSE450 cells.

Cells treated with 15 *μ*M of curcumin were counted (Figure 3Bii) according to morphology (apoptotic, non-apoptotic/MC-like and autophagy-like) and expressed as the percentage of the total cell population. All three morphologies were visible in KYSE450-treated cells, but MC was the predominant morphology (25.4%). KYSE450 cells expressed the highest levels of autophagy-like morphology (22.3%), and the lowest level of apoptosis of all three sensitive cell lines (5.0%). OE21 and OE33 cells show a higher percentage of apoptotic morphology (15.1 and 13.4%, respectively), but MC is more prevalent (25.2 and 22.2%, respectively). OE19 cells show low levels of aberrant morphology. Overall, aberrant morphology counted for ∼50% of the total cell population in sensitive cell lines, which correlates with our initial cell survival data (Figure 1A).

KYSE450 is the most sensitive cell line as evaluated by MTT assay and showed marked cell-cycle arrest in G2/M phase. It is also a cell line where apoptosis appears to be rare after treatment. The dramatic vacuolisation of the cytoplasm without apparent loss of nuclear material is consistent with the described macrostructure of autophagy (F) (Figure 3A) (Kroemer *et al*, 2009). This type of autophagic morphology in the KYSE450 cell line has also been observed by our group in response to treatment with cytotoxic drugs such as 5-fluorouracil and cisplatin (unpublished data). Interestingly, the features of MC and those of autophagy were not obvious in the same cell at the macrostructure level – although it is possible that the vesicles are just smaller in a cell exhibiting MC, or obscured by the dramatic chromatin morphology.

Therefore, this analysis indicates that the primary morphology in susceptible cells resembles that described for MC. The two most susceptible cells lines also show different additional subpopulations. OE21 cells show a minor population with apoptotic morphology and the KYSE450 cells show an additional population with features of autophagy. OE33 and OE19 also show minor populations of apoptosis and autophagy, respectively.

### Examination of caspase-3 activity

**Figure 4:**
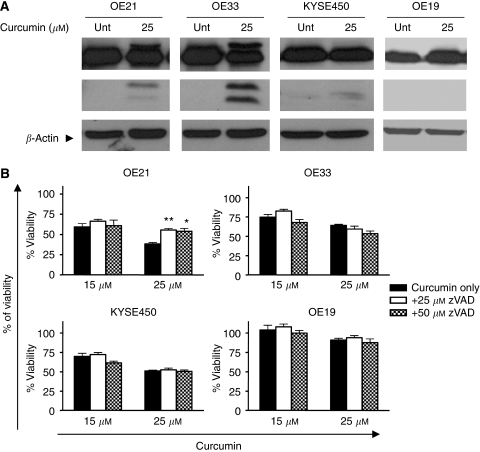
Caspase-3 activity and cell viability after treatment with zVAD and curcumin. (**A**) All four cell lines were treated with 25 *μ*M of curcumin for 24 h. Active caspase-3 activity was estimated by western blotting using equal amounts (40 *μ*g) of whole-cell lysates. *β*-Actin was used as a loading control. The western blot shown is representative of three independent experiments. (**B**) Cell viability estimated by MTT assay in all four oesophageal cancer cell lines after treatment with curcumin and pan-caspase inhibitor zVAD-fmk for 24 h. Data was expressed as the percentage of the control. ^*^P<0.05 and ^**^P<0.005 compared with untreated cells (paired Student's *t*-test). Bars=s.d.

Various papers have suggested that MC is an independent death process, whereas others have proposed that MC is just a consequence of mitotic failure that leads to apoptosis (Castedo *et al*, 2004; Vakifahmetoglu *et al*, 2008). We therefore looked for the presence of active caspase-3, which is an indicator of classical apoptosis. Western blot analysis was used to assess the levels of cleavage of pro-caspase 3 (35 kDa) to active caspase-3 (17 kDa). OE21 and OE33 cell lines showed active caspase-3 after curcumin treatment, consistent with their morphological features (Figure 4A). KYSE450 and OE19 cell lines do not show a significant increase in active caspase-3 with curcumin treatment, which is also consistent with rare apoptotic morphology. It is also notable from the western blot analysis of caspase isoforms that, although active caspase 3 is detectable in OE21 and OE33 cell lines, the majority of the total protein is in the uncleaved pro-caspase form and its contribution to the death process may be relatively minor. We therefore examined the consequences of caspase inhibition on cell viability.

Cells were pretreated with the pan-caspase inhibitor zVAD-fmk and the effect on cell death induced by 25 *μ*M of curcumin was assessed by MTT assay. In OE21 cell lines, the addition of zVAD-fmk at 25 or 50 *μ*M concentration marginally improved only cell viability (Figure 4B), which is consistent with morphological evidence of a minor apoptosis population. In all other cells, the addition of zVAD-fmk does not improve cell viability. These results suggest that a non-apoptotic form of cell death predominates in all of the cell lines with curcumin treatment.

### Induction of autophagy with curcumin treatment

Our results thus far suggest that sensitivity to curcumin is consistent with the induction of MC (with a minor induction of apoptosis in OE21 cells). However, it is clear that another type of cell death coexists in the KYSE450 cell line and, to a lesser extent, in the more resistant OE19 cells. These KYSE450 cells show extensive cytoplasmic vacuolisation, which is consistent with the morphology described for autophagy. Autophagy is a process that sequesters cytoplasmic proteins or organelles into a lytic compartment that facilitates degradation and re-cycling (Xie and Klionsky, 2007). This can promote cell survival during cell stress; however, excessive autophagy can also lead to cell death (Degterev and Yuan, 2008). To investigate whether autophagy coexisted in the cell lines after curcumin treatment, we used the MDC assay, which is a selective fluorescent marker of autophagic vesicles (Biederbick *et al*, 1995). The dye shows diffuse staining in non-autophagic cells, but is sequestered into punctate vesicular staining when autophagy is induced. We therefore examined distribution of the dye in the three curcumin-sensitive cell lines (OE21, OE33 and KYSE450) after treatment with curcumin.

**Figure 5:**
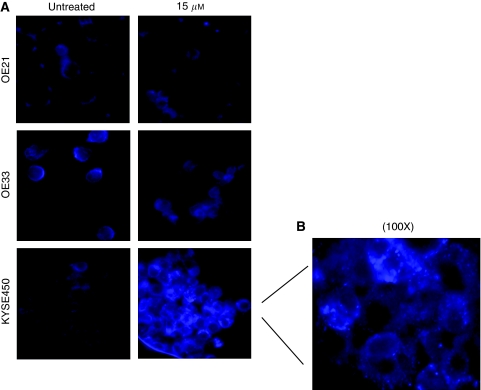
Examination of autophagic cell death in OE21, OE33 and KYSE450 cell lines after treatment with increasing concentrations of curcumin for 24 h. Autophagic vacuoles were identified after incubation of the cells with the selective fluorescent probe monodansylcadaverine (MDC) and visualised immediately by fluorescent microscopy. (**A**) Fluorescent photographic images of curcumin-sensitive cell lines, OE21, OE33 and KYSE450. Images correspond to untreated and 15 *μ*M of curcumin at 24 h after incubation with MDC. Photographs are representative of two different experiments. Original magnification, × 40. (**B**) Detailed photograph of the distinct punctate staining in KYSE450 cells after treatment with 15 *μ*M of curcumin and incubation with MDC. Original magnification, × 100.

OE21 and OE33 cells treated with 15 *μ*M (and 25 *μ*M; data not shown) curcumin show diffuse and non-vesicular staining of MDC (Figure 5A). The KYSE450 cell line has weak and diffuse staining of MDC in untreated cells. However, treated cells show clear punctate/vesicular staining, which is accentuated at the higher curcumin concentration. The punctate staining is clearly visible at the higher magnification of KYSE450 cells (Figure 5B). However, it should be noted that this staining was not universal, and it may coexist and be separate from cells showing MC.

### Curcumin effects on the UPS and cyclin B1

**Figure 6:**
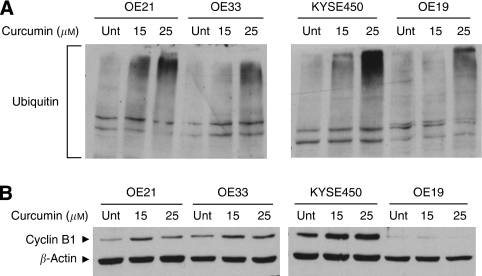
Poly-ubiquitination and cyclin B1 expression in oesophageal cancer cell lines after treatment with increasing concentrations of curcumin for 24 h. (**A**) Effects of curcumin treatment on poly-ubiquitination. Equal amounts (30 *μ*g) of whole-cell lysates were separated using SDS–PAGE and protein poly-ubiquitination assessed by western blotting with an anti-ubiquitin antibody. Accumulation of high-molecular-weight ubiquitin complex is evident after curcumin treatment. (**B**) Cyclin B1 expression in oesophageal squamous and adenocarcinoma cell lines. Equal amounts (30 *μ*g) of whole-cell lysates were separated using SDS–PAGE and protein expression assessed by western blotting with an anti-cyclin B1 antibody. *β*-Actin was used as a loading control. The western blot shown is representative of three independent experiments.

Our data suggest that susceptibility to curcumin is primarily associated with the induction of arrest within G2/M phase and MC. The primary target of curcumin is unknown; however, an emerging body of evidence suggests that this compound may achieve its diverse effects through inhibition of the UPS (Henke *et al*, 1999; Uhle *et al*, 2003; Jana *et al*, 2004; Si *et al*, 2007). We therefore looked for evidence of the accumulation of poly-ubiquitinated proteins after treatment. Cells were treated with 15 and 25 *μ*M of curcumin for 24 h and analysed for the presence of poly-ubiquitinated proteins by western blotting. High-molecular-weight staining increased in a dose-dependent manner – with highest staining evident in the most sensitive cells lines (Figure 6A). Although we currently cannot distinguish this as a mechanism or a consequence of MC, our data are clearly supportive of the UPS contributing a role in the action of this compound.

The UPS is known to have a major role in cell-cycle regulation – particularly at the mitotic checkpoint. CDK1/cyclin B1 accumulates in the G2 phase, at the G2/M border. UPS-mediated degradation of cyclin B1 is then required for cell-cycle progression through mitosis (Kops *et al*, 2005; Vassilev *et al*, 2006). If the UPS system is inhibited in these cell lines, then cyclin B1 is likely to accumulate and may contribute to the major disturbances in mitotic checkpoints. We therefore examined expression after treatment with 15 and 25 *μ*M of curcumin. Cyclin B1 expression increased in a dose-dependent manner after curcumin treatment of KYSE450, OE21 and OE33 cell lines (Figure 6B). The OE19 cell line has low baseline expression of cyclin B1 and does not undergo detectable changes in expression. The elevation in cyclin B1 levels in sensitive cells corresponds with increased levels of protein ubiquitination and induction of arrest within the G2/M phase. It is therefore possible that cyclin B1 elevation contributes to the cell-cycle disturbance that is evident in these cells, but many other proteins are likely to be upregulated as a consequence of disturbances of the UPS and could contribute to the cytotoxicity that follows in the form of MC, apoptosis or autophagy.

## Discussion

Curcumin has shown potential as both a chemopreventive agent and a chemotherapeutic agent in various gastrointestinal cancers, and initial studies in oesophageal cancer have been encouraging (Ushida *et al*, 2000). In this study, we have investigated the effects of curcumin on panel of genetically and phenotypically heterogeneous oesophageal cancer cell lines. We have investigated the cell death mechanisms induced, and also examined additional molecular features associated with cytotoxicity.

Curcumin was shown to inhibit cell proliferation and viability, but susceptibility varied between cell lines. The most sensitive cell lines (KYSE450, OE21 and OE33) underwent mitotic arrest with morphological features of MC (Castedo *et al*, 2004; Vitale *et al*, 2005). The published literature is divided as to whether MC is an event that precedes apoptotic cell death or is a distinct mechanism of cell death (reviewed in Vakifahmetoglu *et al*, 2008). Although morphological analysis did show apoptotic cells in OE21 and OE33, these are coexistent but not dominant features of curcumin-treated cell populations. If apoptosis was the only death mechanism, the low level of apoptosis is not consistent with the complete inability of OE21 cells to recover after treatment with 15 *μ*M of curcumin. The actual death is therefore morphologically distinct from apoptosis. Other authors have reported biochemical features of apoptosis in cell populations showing MC morphology – primarily caspase activation, although indeed with minimal caspase 3 cleavage (Rashmi *et al*, 2004, 2005). Another study also reported that caspase activation was not required for MC and that activation of caspases varied in different cell lines (Mansilla *et al*, 2006). Clearly, limited caspase activation in a heterogeneous population of cells (as in OE21 cells) does not necessarily mean that the MC cells are activating caspases. We have found minimal caspase 3 cleavage in two of our four cell lines (OE21/OE33) and only marginal protective effects of caspase inhibition on viability in one cell line. These results collectively suggest that caspase-dependent apoptosis is not of major importance for curcumin-induced cytotoxicity and that MC may be occurring as an independent death mechanism or is part of an undefined mechanism.

The KYSE450 cell line is particularly sensitive to curcumin and cells show predominantly MC morphology after treatment. However this coexists with cells that show high levels of cytoplasmic vacuolisation, retention of the nuclear membrane and distinct vesicular MDC staining, indicative of autophagy. The morphology suggests that MC did not occur in the same cells as those undergoing autophagy-like cell death, but we cannot rule this out. Other authors have recently reported exclusive autophagic cell death after curcumin treatment and this was associated with the activation of ERK signalling (Aoki *et al*, 2007; Shinojima *et al*, 2007). However, we found de-phosphorylation of ERK after curcumin treatment (Supplementary data). It is possible that this difference is cell-line-related – or dose-related – as our study used much lower concentrations of drug (Aoki *et al* 80 *μ*M and our study 15/25 *μ*M). A number of studies have also linked downregulation of survivin with MC (Magalska *et al*, 2006; Wolanin *et al*, 2006). We did not see a correlation between low survivin expression and induction of MC in the oesophageal cancer cells (Supplementary data).

In this panel of oesophageal cells, the induction of MC was the primary determinant of cytotoxicity. As this can be accompanied by either apoptosis or autophagy – depending on the cell line – the secondary death mechanism must depend on the genetic competency of the cell line, and therefore the induction of either apoptosis or autophagy is not the determinant of sensitivity.

A particularly encouraging aspect to this study is the fact that the induction of MC appears to be a separate distinct death process that could potentially eliminate cancer cells when other death processes fail. The KYSE450 cell line is highly resistant to 5-FU and cisplatin, fails to induce apoptosis but induces primarily an autophagic response (unpublished data). Yet, when treated with curcumin, this cell line is the most sensitive – and death is primarily associated with MC. The OE19 cell line is also apoptosis-incompetent in response to cisplatin and 5-FU – and will undergo MC in response to curcumin – although at much higher concentrations (data not shown). The clinical implications of these results are clear: it is possible that one of the factors affecting poor response to chemotherapy is the presence of ‘apoptosis-incompetent’ cells. The ability to undergo autophagy may not necessarily be helpful, as it can contribute to cell survival. The existence of MC as an alternative cell death pathway may therefore offer another possibility for treatment. Curcumin or other inducers of MC may represent important classes of chemotherapeutics for apoptosis-incompetent cells.

Despite evidence of curcumin's potential as an anticancer cytotoxic agent, we still have little understanding of the mechanism: clearly, there are different effects on different cell types. Curcumin has the capacity to be biphasic in its effects: it can be used to promote wound healing, yet be cytotoxic. This would suggest that either it has multiple targets that are expressed in a cell-specific manner or a target that affects cell-specific pathways. We report in this study that curcumin treatment leads to the accumulation of poly-ubiquitinated proteins. This was consistent with cyclin B1 upregulation and G2/M arrest. Our data are therefore supportive of the view that curcumin acts as an inhibitor of the UPS (Henke *et al*, 1999; Jana *et al*, 2004; Si *et al*, 2007). This is likely to have marked effects on the cell cycle, but effects may differ in different cell lines because of differences in their existing proteome. For example, cells of a similar type that have lost transcription of tumour suppressors and/or other cell cycle inhibitors may differ in their response. Another reported activity of curcumin that may explain its pleiotropic nature is its effects on histone modification (Liu *et al*, 2005). We cannot rule this out, and clearly more mechanistic work needs to be undertaken in this area. It is also noteworthy that the UPS system can influence chromatin structure and thus transcription (O’Connell and Harper, 2007).

In current cancer treatment protocols, the ‘cell death competency’ of tumours is always undetermined and for many inaccessible cancers this may never change. It is possible that chemopreventive or chemotherapeutic regimes may be most effective if they targeted several death pathways in cancer cells. At least three different Phase I clinical trials have indicated that curcumin is safe and well tolerated even at doses as high as 12 g day^−1^ (Cheng *et al*, 2001). However, despite its well-documented efficacy and safety in both animals and humans *in vivo* and *in vitro*, its poor bioavailability has been cited as a major concern and has probably delayed its development as a therapeutic agent. Serum concentrations of curcumin after oral administration range from 0.013 to 11.1 *μ*M (Anand *et al*, 2007). Various studies have shown curcumin has low bioavailability, limited tissue distribution, short half-life and a rapid metabolism irrespective of its route of administration (Ravindranath and Chandrasekhara, 1981; Garcea *et al*, 2004, 2005; Hoehle *et al*, 2006; Anand *et al*, 2007). The fact that curcumin has poor systemic bioavailability would suggest that the gastrointestinal tract is the most promising site for use in prevention or therapy. However, novel formulations and metabolism inhibitors attempting to optimise the pharmacological potency of curcumin have gained attention, such as the use of piperine, nanoparticles, liposomes, phospholipid complexes and structurally altered derivatives and analogues that dramatically improve the biological activity of curcumin (Shoba *et al*, 1998; Mishra *et al*, 2005; Liu *et al*, 2006; Anand *et al*, 2007; Li *et al*, 2007; Mosley *et al*, 2007). Combination studies so far in both *in vitro* and *in vivo* have highlighted promising combinations of curcumin with oxaliplatin (Howells *et al*, 2007; Li *et al*, 2007; Patel *et al*, 2008) and paclitaxel (Aggarwal *et al*, 2005), suggesting there is a very definite potential for curcumin to be used as part of mainstream clinical regimes.

In summary, curcumin shows features of cytotoxicity, predominantly consistent with the induction of MC in sensitive cell lines. This can be accompanied by features of apoptosis or autophagy, depending on the cell line. As this drug clearly has activity in apoptosis-resistant cells, it is likely that curcumin and indeed some of its bioavailability-enhanced analogues are realistic options to be considered in the future for targeted molecular cancer prevention and treatment.
