# Supplementary figures and images for: Curcumin induces apoptosis-independent death in oesophageal cancer cells
Source: Br J Cancer. 2009 Oct 6;101(9):1585–95. doi: 10.1038/sj.bjc.6605308 (PMC2778521; doi:10.1038/sj.bjc.6605308)

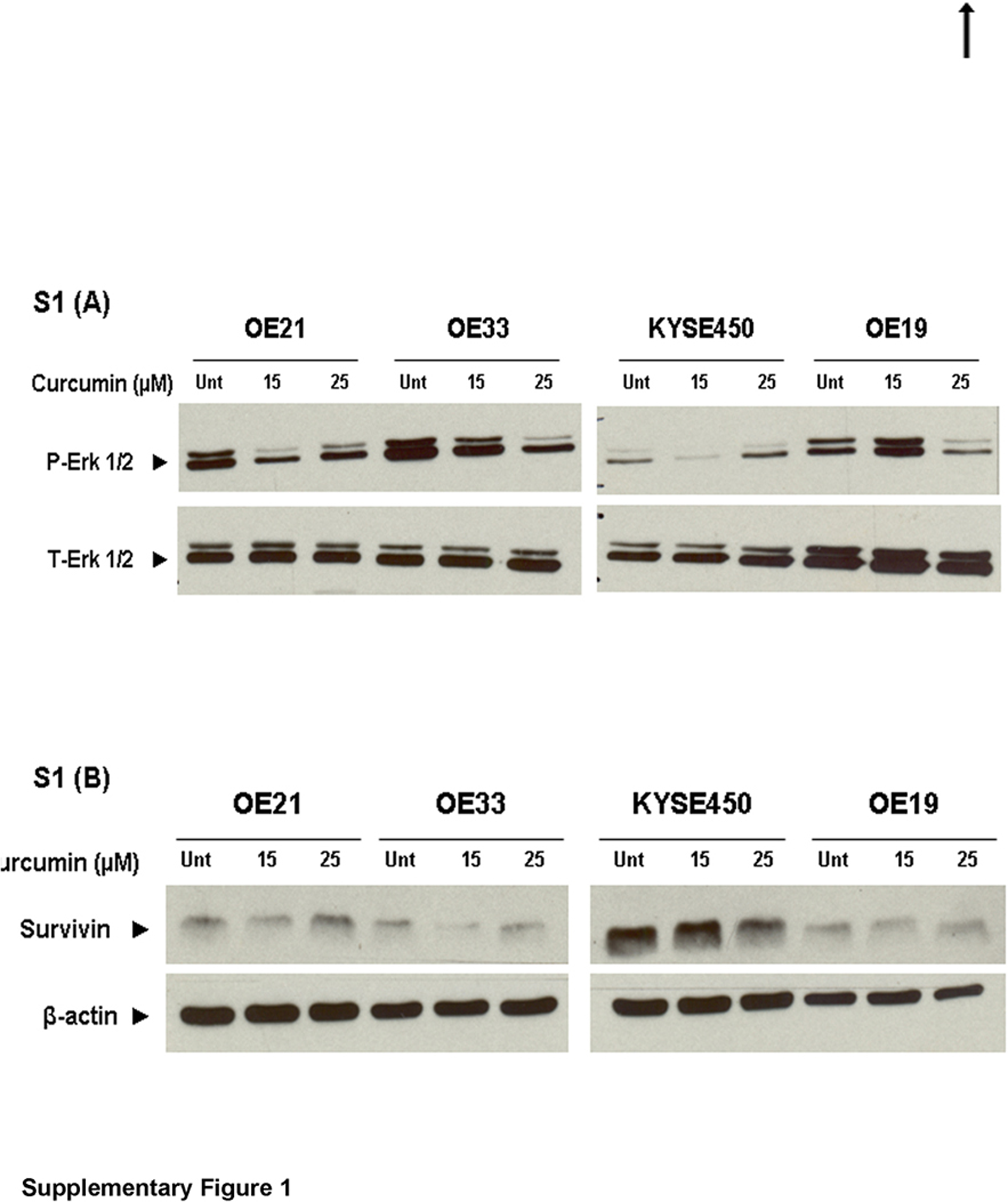

Supplement: Supplementary Figure 1 [file 6605308x1.tif]
