# Supplementary material for: Curcumin induces apoptosis-independent death in oesophageal cancer cells
Source: Br J Cancer. 2009 Oct 6;101(9):1585–95. doi: 10.1038/sj.bjc.6605308 (PMC2778521; doi:10.1038/sj.bjc.6605308)
Supplement: Supplementary data [file 6605308x2.doc]

**Supplementary data**

*Effect on MAP kinases and survivin*

A recent study has reported induction of autophagy by curcumin in glioma cells (12), and suggested that activation of MAP kinases (elevated phospho-Erk) may be responsible for autophagy induction. We examined the status of Erk phosphorylation in the oesophageal cancer cells following treatment with curcumin, however we found that Erk phosphorylation decreased in treated cells (Fig. S1(A)). This occurred irrespective of apoptosis or autophagy induction, as Erk was inhibited in all sensitive cells. Other studies have reported that survivin protein expression decreases significantly after treatment with curcumin, and link this down-regulation with the mechanism behind the morphological features of mitotic catastrophe (10, 11). We found no correlation between low survivin expression and induction of MC (Fig. S1(B)).

**Figure Legends**

**Figure S1(A)** ERK phosphorylation in oesophageal cancer cell lines.

ERK phosphorylation was assessed in oesophageal cancer cell lines following treatment with increasing concentrations of curcumin over 24 hours. Equal amounts (30µg) of whole cell lysates were separated using SDS-PAGE and protein expression assessed by Western blotting with anti-phospho Erk. Total-ERK was used as a loading control. This data indicates decreased phosphorylation of Erk following curcumin treatment.

**Figure S1(B).** Survivin protein expression following curcumin treatment.

Survivin expression was investigated in all four cell lines following 24 hours of treatment with curcumin. Equal amounts (60µg) of whole cell lysates were prepared from untreated control cells and cells treated with increasing concentrations of curcumin for 24 hours. β-actin was used as a loading control. The Western blot shown is representative of three independent experiments. In the OE21 and OE33 cell lines, survivin protein was decreased following treatment with 15µM of curcumin but this was not consistent at 25mM, which induces significant cell cycle arrest and is more cytotoxic. In the KYSE450 cell line, survivin protein expression was high and decreased at 25μM curcumin. However expression levels are still relatively high compared to cells that are less sensitive to the drug. Survivin expression in OE19 cells did not vary significantly from baseline levels. Therefore while down-regulation of survivin may accompany MC in a given cell line treated with a specific concentration – we have found no correlation between low survivin expression and induction of mitotic catastrophe.
